# Changes in Fasting plasma glucose status and risk of mortality events in individuals without diabetes over two decades of Follow-up: a pooled cohort analysis

**DOI:** 10.1186/s12933-022-01709-z

**Published:** 2022-12-03

**Authors:** Karim Kohansal, Soroush Masrouri, Davood Khalili, Azra Ramezankhani, Fereidoun Azizi, Michael J Blaha, Farzad Hadaegh

**Affiliations:** 1 Prevention of Metabolic Disorders Research Center, Research Institute for Endocrine Sciences, Shahid Beheshti University of Medical Sciences, Tehran, Iran; 2 Endocrine Research Center, Research Institute for Endocrine Sciences, Shahid Beheshti University of Medical Sciences, Tehran, Iran; 3 Department of Cardiology, Johns Hopkins Ciccarone Center for the Prevention of Cardiovascular Disease, Johns Hopkins University, Baltimore, MD USA

**Keywords:** All-cause, cancer, Cardiovascular, Fasting plasma glucose, Men, Mortality, Women

## Abstract

**Background:**

We aimed to assess the gender-specific impact of 3-year changes in fasting plasma glucose (FPG) status on the risk of all-cause, cardiovascular (CV), and cancer mortality in individuals without type 2 diabetes (T2DM) during an 18-year follow-up.

**Methods:**

The study population included 14,378 participants aged 30–60 years (8272 women) from three population-based cohort studies, including Atherosclerosis Risk in Communities, Multi-Ethnic Study of Atherosclerosis, and Tehran Lipid and Glucose Study. Subjects were classified into six categories based on the approximately three-year changes in FPG status: (1) normal FPG (NFG) to NFG (reference category); (2) NFG to impaired fasting glucose (IFG) (i.e., 126 > FPG ≥ 100 mg/dl); (3) NFG to T2DM; (4) IFG to NFG; (5) IFG to IFG; (6) IFG to T2DM. Multivariable stratified Cox regression, adjusting for age, body mass index (BMI), BMI-Change, smoking status, hypertension, and hypercholesterolemia was used to estimate hazard ratios (HRs (95% CI)) for all-cause and cause-specific mortality events. Women-to-men ratios of HRs (RHRs) for each category were also estimated.

**Results:**

During follow-up, 2,362 all-cause mortality events were recorded. Among women, all categories of FPG change, excluding IFG-NFG (HR, 95%CI 1.24 (0.98–1.57), p = 0.07), were associated with a higher risk of all-cause mortality compared to the NFG-NFG category. Moreover, women in IFG-T2DM group were at increased risk for CV mortality (2.21 (1.42–3.44)). We also found that women in NFG-IFG (1.52 (1.20–1.91)), NFG-T2DM (2.90 (1.52–5.51)), and IFG-IFG (1.30 (1.02–1.66)) categories had a higher risk for cancer mortality. However, among men, a higher risk of all-cause mortality was found for only two groups of NFG-T2DM (1.78 (1.15–2.74)) and IFG-T2DM (1.34 (1.04–1.72)). Women with IFG-IFG had a 24% higher risk for all-cause mortality events than their men counterparts (RHR; 1.24 (1.01–1.54)). After further adjustment for physical activity, results were in line with the main findings, excluding T2DM up to six years after the measurement period and early mortality events.

**Conclusion:**

In women, the IFG status, whether as incident, persistent, or converted to T2DM, had a higher risk for mortality events; however, among men, only conversion to T2DM conferred an excess risk of all-cause mortality.

**Supplementary Information:**

The online version contains supplementary material available at 10.1186/s12933-022-01709-z.

## Background

Fasting plasma glucose (FPG) measurement is widely used for detecting type 2 diabetes (T2DM) and pre-diabetes in clinical practice because it is less time-consuming and inexpensive compared to the alternatives, and it is available worldwide [1]. Studies have shown that higher FPG levels are associated with excess risk of mortality and cardiovascular disease (CVD) in individuals without T2DM [2–5] and introduced it as an independent predictor for cardiovascular (CV) and all-cause mortality [2, 6]. It is worth noting that most of the published studies on this topic are based on a single measurement of FPG and do not consider the potential effects of changes in FPG concentrations over time.

Not accounting for the time-varying of FPG status could be one critical missing thread in the studies investigating the effects of glycemic status on mortality. A Finnish study showed that impaired glucose tolerance (IGT) could cause an increased risk of CV and all-cause mortality independent of conversion to T2DM [7]. In another study among Korean people, incident IFG in normoglycemic subjects was associated with a higher mortality risk; however, the researchers did not show whether this risk was independent of the conversion to T2DM [8]. As far as we know, very few studies have investigated the effects of changes in FPG status on all-cause mortality [8–10] or CV mortality [11]. In the Iranian population, we previously showed that among participants with pre-diabetes at baseline, only those who developed T2DM had a higher risk of CVD [12]. Considering mortality events, to our knowledge, only one US-based study showed that the unfavorable impact of impaired fasting glucose (IFG) on mortality events was mainly due to the conversion to T2DM [11]. Moreover, It is not entirely clear whether the long-term health risks associated with IFG are due to mildly elevated plasma glucose levels or to future progression to diabetes [6].

Growing evidence also supports the existence of significant sex differences in morbidity- and mortality-related complications of T2DM. Previous studies suggest that T2DM is a more decisive risk factor for vascular disease and mortality in women than in men [13, 14]. These findings are still debated in the non-diabetic population, and firm conclusions have not yet been reached. All of the above indicate the need for studies examining the impact of changes in FGP status on hard outcomes, evaluating the effect modification of gender in the non-diabetic population. In this study, we used pooled analysis of three large cohort studies and aimed to investigate the sex-specific impact of short-term changes in FPG status on the long-term risk of all-cause, CV, and cancer mortality in individuals without T2DM at baseline. We also aimed to compare the impacts of short-term FPG changes in women versus men on mortality events.

## Methods

### Study population

The present study included individual-level data from three population-based cohort studies designed to study the risk factors of T2DM and cardiovascular disease: Atherosclerosis Risk in Communities (ARIC), Multi-Ethnic Study of Atherosclerosis (MESA), and Tehran Lipid and Glucose Study (TLGS). ARIC and MESA public-use datasets were obtained through the National Heart, Lung, and Blood Institute (NHLBI) Biologic Specimen and Data Repository Information Coordinating Center (BioLINCC). The study design, objectives, and participant characteristics for each cohort have been previously described [15–18] and presented briefly in Additional file 1.

**Fig. 1:**
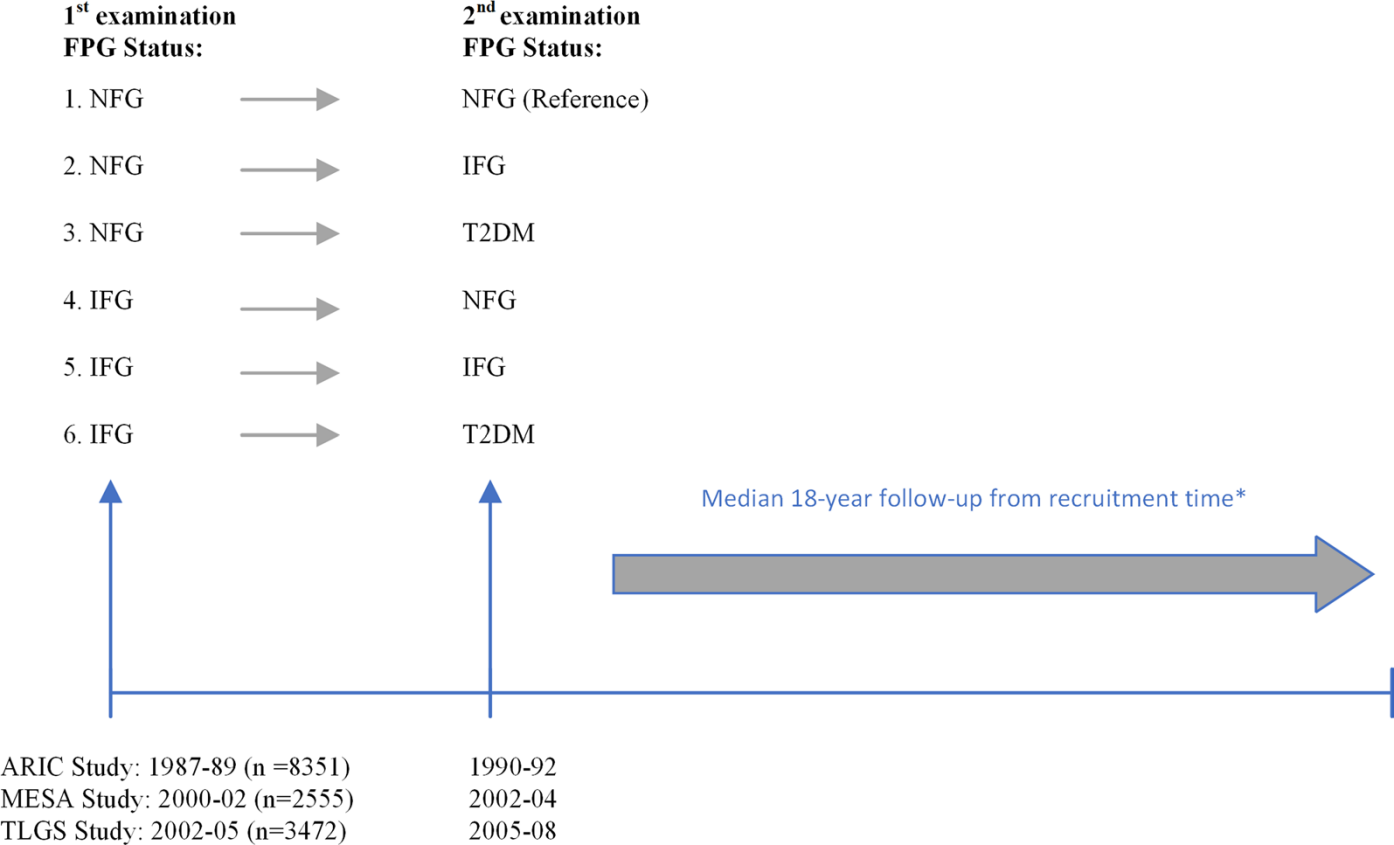
Timeline of the study design. Subjects without diabetes were followed from the first examination to the second examination for the fasting plasma glucose (FPG) changes [1] Normal fasting glucose (NFG) to NFG, [2] NFG to impaired fasting glucose (IFG), [3] NFG to type 2 diabetes mellitus (T2DM), [4] IFG to NFG, [5] IFG to IFG [6] IFG to T2DM. *Only ARIC and TLGS participants were assessed for cancer mortality outcomes and followed-up for a median of 25.1 years

For the present analysis, a total of 20,822 individuals aged ≤ 60 and ≥ 30 years from the initial phases of ARIC (1987–89) (n = 12,162), MESA (2000–02) (n = 3100), and phase two for TLGS (2002–05) (n = 5560) cohorts were considered as the baseline population (Additional file 1: Fig. S1). The next phase for all studies was considered as the second examination to determine short-term changes in FPG status. After excluding those with prevalent CVD at baseline or incident CVD events before the second examination (n = 1276) and those with T2DM (n = 1434) at baseline, 18,112 individuals remained. Other exclusion criteria included those who did not participate in the second examination (n = 841), and those with missing data on covariates at baseline (including body mass index (BMI), smoking, FPG, systolic and diastolic blood pressure (SBP, DBP), total cholesterol (TC), taking antihypertensive, lipid-lowering, and blood glucose-lowering drugs) and second examination (including BMI, FPG, and using blood glucose-lowering drugs) (n = 2893, considering overlap features between numbers), leading to a dataset with 14,378 participants for all-cause and CV mortality (polling all three cohorts) and 11,823 participants for cancer mortality (pooling ARIC and TLGS). The timeline of the study design for respondents of three cohort studies is represented in Fig. 1.

### Definition of variables

T2DM was defined as using blood glucose-lowering agents or FPG ≥ 7 mmol/L (126 mg/dl). IFG was defined using diagnostic criteria by the American Diabetes Association (ADA) and WHO: (1) ADA: FPG ≥ 5.6 mmol/L (100 mg/dl) and < 7 mmol/L (126 mg/dl) without using glucose-lowering agents, (2) WHO: FPG ≥ 6.1 mmol/L (110 mg/dl) and < 7 mmol/L (126 mg/dl) without using blood glucose-lowering agents. Hypertension was defined as SBP ≥ 140 mmHg or DBP ≥ 90 mmHg, or the use of antihypertensive medications. Hypercholesterolemia was determined by TC ≥ 5.17 mmol/L (200 mg/dl) or the use of lipid-lowering drugs. BMI was defined as weight in kilograms divided by height in meters squared (kg/m^2^). BMI-Change was calculated as (Second examination BMI measurement) – (First examination BMI measurement). CVD at baseline was defined as a history of heart failure (HF), myocardial infarction (MI), coronary revascularization, stroke, or transient ischemic attack. Based on approximately three-year (median of 2.85 and standard deviation (SD) of 0.66 years) changes in FPG status, subjects were classified into six mutually exclusive categories: (1) normal fasting glucose (NFG) to NFG (reference category in all analyses), (2) NFG to IFG, (3) NFG to T2DM (4) IFG to NFG, (5) IFG to IFG, (6) IFG to T2DM.

### Outcomes

#### ARIC

The cause of death for study participants was ascertained through annual cohort follow-up, community-wide hospital surveillance, and linkage with local and national death registries. The date and cause of death were verified by death certificate review. All-cause mortality was defined as death from any cause. CV mortality was defined as any death in which the principal cause was CV, using the ICD-9 codes 390-459 or ICD-10 codes I00-I99. Cancer mortality was defined using the ICD-9 codes 140-208 or ICD-10 codes C00-C97 [17].

#### MESA

At 9–12 months intervals, a telephone interviewer inquired about interim hospital admissions, CV diagnoses, and deaths. An adjudication committee received copies of all death certificates and medical records for hospitalizations and outpatient CV diagnoses and conducted next-of-kin interviews for out-of-hospital CV deaths for verification. Records were obtained on 98% of reported hospitalized CVD events. Two physicians independently classified and assigned incidence dates. A full mortality and morbidity review committee made the final disagreement classification [19]. A more detailed description of the MESA follow-up methods is available at http://www.mesanhlbi.org.

#### TLGS

To summarize, a trained nurse interviewed participants for any new medical events through an annual phone call. In cases of mortality, related data were collected by a trained physician using hospital records and/or home visits. In the case of mortality outside the hospital, data were collected from the death certificate and forensic medicine report. A verbal autopsy was performed using a standard questionnaire which consisted of the time and location (in-home or hospital) of death, medical events, or complications leading to death. Collected data were then evaluated by an outcome committee consisting of an internist, endocrinologist, cardiologist, epidemiologist, and other experts, when needed, to assign a specific outcome for every event [20, 21]. For the current study, all-cause mortality (defined as death from any cause) and specific mortalities including CV (ICD-9 codes 390-459, or ICD-10 codes I00-I99) and cancer (ICD-9 codes 140-208, or ICD-10 codes C00-C97) were considered.

### Statistical analysis

Descriptive statistics in mean (SD) and number (percentage) were generated for participants’ characteristics in the overall cohort and compared between the six categories of changing FPG status separately in men and women. Moreover, a comparison between respondents (study participants) and non-respondents (subjects with missing data or lost to follow-up) was conducted. Continuous variables were compared using the ANOVA and t-test, as appropriate. Categorical variables were compared by the chi-square test.

The crude incidence rates and 95% confidence interval (95% CI) per 1000 person-years were calculated for each outcome.

Cox proportional hazard regression models were used to estimate hazard ratios (HRs) of different categories of change in FPG status for mortality events, considering those in NFG-NFG group as the reference category. Time to event was defined by the time of censoring or the event occurring, whichever came first. Stratified baseline hazards were used to account for the study-to-study heterogeneity that may be caused by differences in the cohort studies’ populations or data collection methods. Cox regression models were adjusted for known risk factors at baseline, including age, BMI, BMI-Change, hypertension, hypercholesterolemia, and smoking status. In all models, we included an interaction term of FPG changing groups with sex to estimate the women-to-men ratios of HRs (RHRs) for each category. The proportional hazards assumption in the Cox model was assessed using Schoenfeld residual test, and all proportionality assumptions were appropriate. All statistical analyses were performed using R version 3.6.2, and 2-sided p-values < 0.05 were considered statistically significant.

## Results

### Descriptive results

The study population included 14,378 people (8272 women) with a mean (SD) age of 50 (6.9) years. Additional file 1: Table S1 shows the baseline characteristics of respondents and non-respondents. Overall, respondents were older but healthier for the other baseline characteristics than non-respondents. Baseline characteristics of individuals in each study group stratified by gender are summarized and compared in Tables 1 and 2. There were significant differences in baseline characteristics in different study groups. Overall, the group of participants who remained normoglycemic on both examinations (NFG-NFG) was in better cardiometabolic state.

**Table 1:** Clinical characteristics according to short-term changes in FPG status in women

Variables	All (n = 8272)	NFG to NFG (n = 5092)	NFG to IFG (n = 1233)	NFG to T2DM (n = 107)	IFG to NFG (n = 445)	IFG to IFG (n = 1160)	IFG to T2DM (n = 235)
Continuous variables, mean ± SD							
Age (years)	49.9 ± 6.9	48.8 ± 7.4	51.5 ± 5.3	50.5 ± 6.8	51.0 ± 6.5	52.2 ± 5.2	51.0 ± 6.1
SBP (mmHg)	116 ± 17.6	113 ± 16.6	118.2 ± 17.5	123.5 ± 17.3	120.1 ± 18.4	122.4 ± 18.2	125.7 ± 19.5
DBP (mmHg)	72.1 ± 10.5	70.9 ± 10.3	72.8 ± 10.5	74.7 ± 11.1	74.8 ± 10.4	74.4 ± 10.1	76.6 ± 11.2
BMI (kg/m^2^)	27.9 ± 5.7	26.9 ± 5.1	28.4 ± 5.9	32.5 ± 6.2	28.5 ± 5.8	29.7 ± 6.3	32.6 ± 6.6
TC (mmol/L)	5.4 ± 1.1	5.3 ± 1.0	5.5 ± 1.1	5.4 ± 1.2	5.6 ± 1.1	5.7 ± 1.0	5.6 ± 1.2
FPG (mmol/L)	5.2 ± 0.6	4.9 ± 0.4	5.2 ± 0.3	5.7 ± 0.7	5.8 ± 0.2	6.0 ± 0.3	6.3 ± 0.4
Categorical variables, number (%)							
Ever smoker	2976 (36)	1677 (32.9)	521 (42.3)	33 (30.8)	161 (36.2)	496 (42.8)	88 (37.4)
Hypertension (yes)	1995 (24.1)	905 (17.8)	341 (27.7)	43 (40.2)	139 (31.2)	444 (38.3)	123 (52.3)
Hypercholesterolemia (yes)	4546 (55)	2559 (50.3)	727 (59)	60 (56.1)	273 (61.3)	785 (67.7)	142 (60.4)
Outcomes, the incidence rate per 1000 person-years (95%CI)							
Cancer Mortality*	3.34 (3.05–3.65)	2.43 (2.11–2.80)	4.57 (3.78–5.47)	6.64 (3.18–12.21)	4.15 (2.92–5.72)	4.36 (3.58–5.26)	4.34 (2.65–6.70)
CV Mortality	1.85 (1.65–2.08)	1.25 (1.03–1.50)	2.41 (1.86–3.08)	2.31 (0.63–5.91)	2.19 (1.34–3.38)	2.61 (2.02–3.32)	5.43 (3.55–7.95)
All-cause Mortality	7.35 (6.93–7.79)	5.38 (4.91–5.88)	9.53 (8.39–10.78)	11.54 (7.05–17.83)	9.31 (7.44–11.51)	10.05 (8.85–11.36)	12.73 (9.74–16.36)

^*^Only ARIC and TLGS participants were assessed and followed-up for cancer mortality outcomes

As appropriate, baseline data are shown as mean ± standard deviation (SD) or number (percent). Outcome data are shown as incidence rate per 1000 person-years (95% CI)

*NFG* normal fasting glucose, *IFG* impaired fasting glucose based on ADA cut-offs (100 mg/dl), *T2DM* type 2 diabetes mellitus; SBP: systolic blood pressure, *DBP* diastolic blood pressure, *BMI* body mass index, *FPG* fasting plasma glucose, *TC* total cholesterol, *CV* cardiovascular, *CI* confidence interval

**Table 2:** Clinical characteristics according to short-term changes in FPG status in men

Variables	All (n = 6106)	NFG to NFG (n = 2996)	NFG to IFG (n = 1102)	NFG to T2DM (n = 74)	IFG to NFG (n = 417)	IFG to IFG (n = 1318)	IFG to T2DM (n = 199)
Continuous variables, mean ± SD							
Age (years)	50.1 ± 6.9	48.6 ± 7.8	51.3 ± 5.5	49.3 ± 7.1	50.4 ± 6.5	52.0 ± 5.2	52.4 ± 5.6
SBP (mmHg)	118.6 ± 15.6	116.4 ± 15	119.4 ± 15.8	120.6 ± 13.6	120.0 ± 16.3	121.3 ± 15.3	126 ± 17.1
DBP (mmHg)	75.5 ± 10.3	74.8 ± 10	75.8 ± 10.5	77.3 ± 8.6	75.8 ± 11.4	76.3 ± 10.2	78.4 ± 11.2
BMI (kg/m^2^)	27.3 ± 4	26.7 ± 3.8	27.5 ± 4	30.1 ± 4.7	27.5 ± 4.1	28 ± 4	30 ± 4.3
TC (mmol/L)	5.2 ± 1	5.1 ± 1	5.3 ± 1	5.1 ± 1.1	5.3 ± 1	5.4 ± 1	5.4 ± 1
FPG (mmol/L)	5.3 ± 0.6	4.9 ± 0.4	5.3 ± 0.3	5.8 ± 0.7	5.8 ± 0.3	6.0 ± 0.3	6.4 ± 0.4
Categorical variables, number (%)							
Ever smoker	3706 (60.7)	1643 (54.8)	711 (64.5)	51 (68.9)	264 (63.3)	897 (68.1)	140 (70.4)
Hypertension (yes)	1403 (23)	536 (17.9)	265 (24)	21 (28.4)	97 (23.3)	414 (31.4)	70 (35.2)
Hypercholesterolemia (yes)	3104 (50.8)	1368 (45.7)	587 (53.3)	35 (47.3)	215 (51.6)	784 (59.5)	115 (57.8)
Outcomes, the incidence rate per 1000 person-years (95%CI)							
Cancer Mortality*	4.33 (3.93–4.75)	3.05 (2.53–3.65)	5.26 (4.34–6.32)	4.68 (1.52–10.93)	4.80 (3.38–6.62)	4.99 (4.20–5.89)	5.96 (3.78–8.95)
CV Mortality	2.92 (2.61–3.25)	1.97 (1.59–2.42)	3.41 (2.69–4.26)	4.92 (1.81–10.71)	2.86 (1.81–4.29)	3.56 (2.90–4.32)	6.02 (3.81–9.03)
All-cause Mortality	10.82 (10.21–11.44)	7.57 (6.80–8.40)	12.85 (11.42–14.41)	18.05 (11.31–27.32)	11.43 (9.22–14.02)	12.84 (11.56–14.22)	19.07 (15.02–23.86)

^*^Only ARIC and TLGS participants were assessed and followed-up for cancer mortality outcomes

As appropriate, baseline data are shown as mean ± standard deviation (SD) or number (percent). Outcome data are shown as incidence rate per 1000 person-years (95% CI)

*NFG* normal fasting glucose, *IFG* impaired fasting glucose based on ADA cut-offs (100 mg/dl), *T2DM* type 2 diabetes mellitus, *SBP* systolic blood pressure, *DBP* diastolic blood pressure, *BMI* body mass index, *FPG* fasting plasma glucose, *TC* total cholesterol, *CV* cardiovascular, *CI* confidence interval

During a median follow-up (interquartile range (IQR)) of 18 years (95% CI 13.1–26), 2362 (1157 women) and 617 (292 women) incident cases of all-cause death and CV death occurred, respectively. All-cause death rates were 7.35 (6.93–7.79) and 10.82 (10.21–11.44) per 1000 person-year in women and men, respectively. The corresponding values for CV mortality were 1.85 (1.65–2.08) and 2.92 (2.61–3.25) per 1000 person-year. Moreover, among the pooled analysis of ARIC and TLGS (n = 11,823), during a median follow-up (IQR) of 25 (14.9–26.3) years, we found 488 and 439 cancer mortality events with an incidence rate of 3.34 (3.05–3.65) and 4.33 (3.93–4.75) in women and men, respectively.

Kaplan-Meier survival curves for the cumulative incidence of all-cause, CV, and cancer death in six categories of study, separately in men and women, were plotted and represented in the Additional file 1: Figs. S2**–**S4** (**log-rank tests’ p-values < 0.001 in all comparisons).

### Hazards for all-cause mortality

**Fig. 2:**
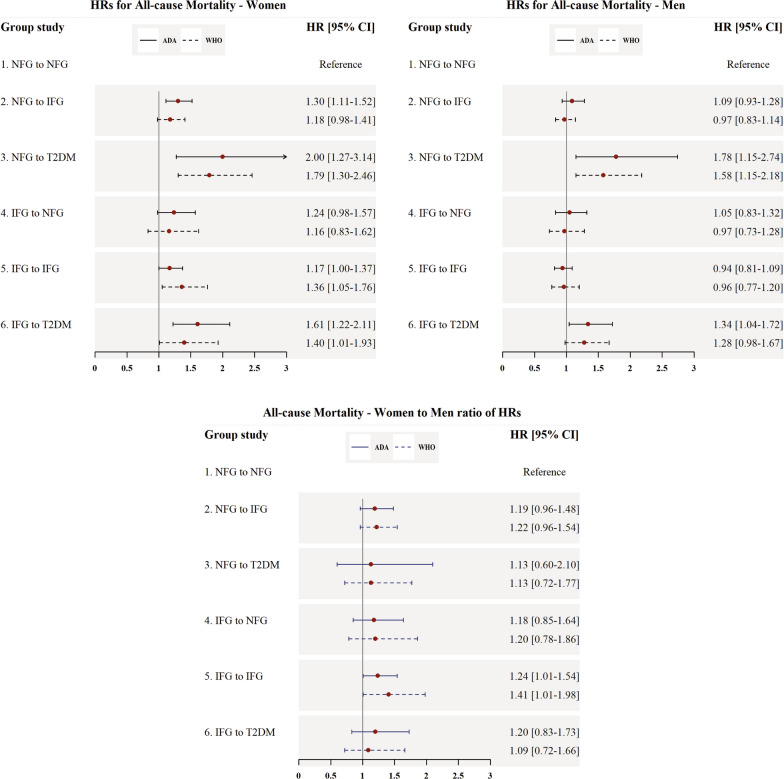
Multivariable adjusted HRs for the incidence of all-cause mortality associated with different categories of fasting plasma glucose changes. *HR* hazard ratio, *CI* confidence interval, *ADA* American Diabetes Association, *WHO* World Health Organization, *NFG* normal fasting glucose, *IFG* impaired fasting glucose based on WHO and ADA cut-offs (100 and 110 mg/dl, respectively), *T2DM* type 2 diabetes mellitus, *BMI* body mass index. All models were adjusted for age, BMI, BMI-Change, smoking status, hypertension, and hypercholesterolemia

Figure 2 shows the association of changes in FPG status with all-cause mortality. Based on the IFG-ADA, in men, participants who converted to T2DM (NFG to T2DM, IFG to T2DM) had about 78% and 34% higher risk for all-cause death, respectively, compared with men in NFG-NFG as the reference. However, considering IFG-WHO, only men who converted from NFG to T2DM showed a significant HR (1.58; 1.15–2.18).

Among women, using IFG-ADA, all categories of FPG change, excluding IFG-NFG (1.24; 0.98–1.57, p = 0.07), showed significant risks for all-cause mortality; the increased HRs ranged from 17% for IFG-IFG to 100% for NFG-T2DM category. However, considering IFG-WHO, women in three groups of NFG-T2DM, IFG-IFG, and IFG-T2DM showed a higher risk for all-cause mortality with HRs of 1.79 (1.30–2.46), 1.36 (1.05–1.76) and 1.40 (1.01–1.93), respectively.

Generally, compared to men, women had a higher but not statistically significant risk for all-cause mortality events in all categories of FPG changes compared to those remaining in NFG. Women who remained in IFG status, compared to the men, had a significant 24% (1.24; 1.01–1.54) and 41% (1.41; 1.01–1.98) higher risk for all-cause mortality using IFG-ADA and IFG-WHO cut-offs, respectively.

### Hazards for CV and cancer mortality

**Fig. 3:**
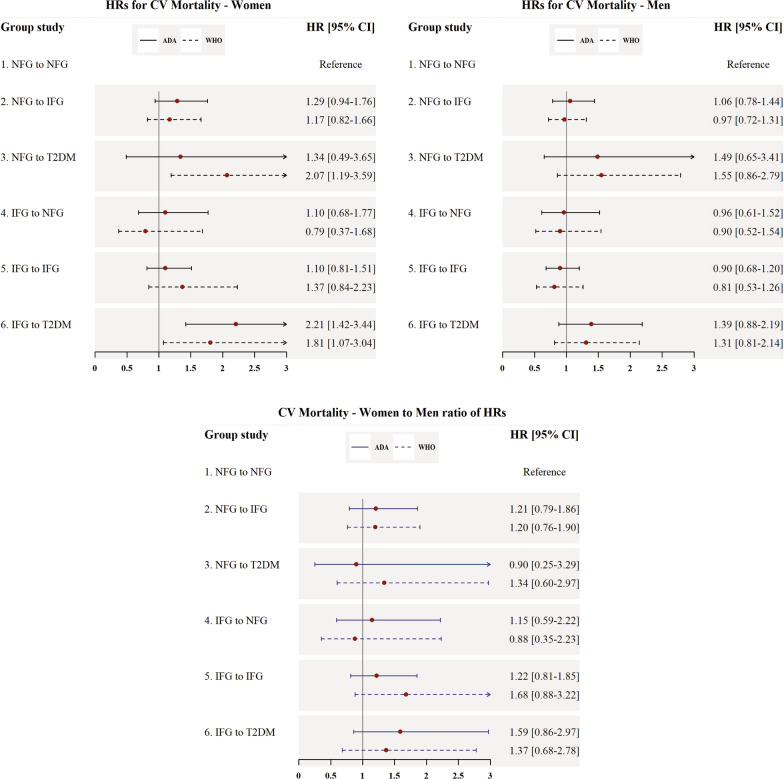
Multivariable adjusted HRs for the incidence of CV mortality associated with different categories of fasting plasma glucose changes. *HR* hazard ratio, *CI* confidence interval, *ADA* American Diabetes Association, *WHO* World Health Organization, *NFG* normal fasting glucose, *IFG* impaired fasting glucose based on WHO and ADA cut-offs (100 and 110 mg/dl, respectively), *CV* cardiovascular, *T2DM* type 2 diabetes mellitus, *BMI* body mass index. All models were adjusted for age, BMI, BMI-Change, smoking status, hypertension, and hypercholesterolemia

**Fig. 4:**
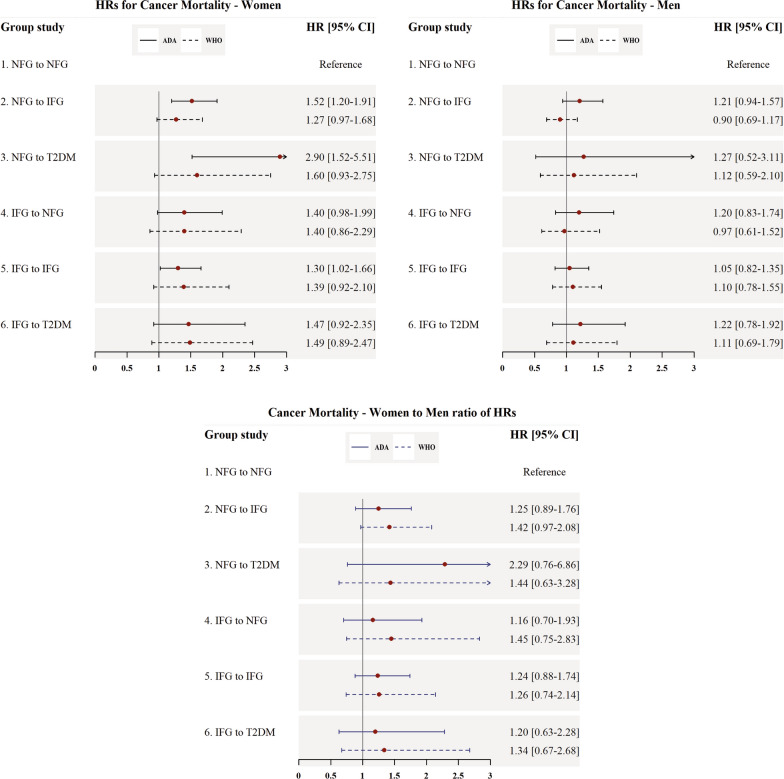
Multivariable adjusted HRs for the incidence of cancer mortality associated with different categories of fasting plasma glucose changes. *HR* hazard ratio, *CI* confidence interval, *ADA* American Diabetes Association, *WHO* World Health Organization, *NFG* normal fasting glucose, *IFG* impaired fasting glucose based on WHO and ADA cut-offs (100 and 110 mg/dl, respectively), *T2DM* type 2 diabetes mellitus, *BMI* body mass index. All models were adjusted for age, BMI, BMI-Change, smoking status, hypertension, and hypercholesterolemia. Cancer mortality was assessed only among participants from ARIC and TLGS studies

Figures 3 and 4 show the HRs of CV and cancer mortality in men and women. Among men, no significant association was found between FPG change and CV mortality. However, among women, subjects who converted from IFG to T2DM showed a significant risk for CV mortality using both IFG-ADA (2.21; 1.42–3.44) and IFG-WHO (1.81; 1.07–3.04) definition. Moreover, using IFG-WHO, conversion from NFG to T2DM in women increased the risk of CV mortality by more than 100% compared to the reference group (2.07; 1.19–3.59).

Considering cancer mortality, all of the categories of FPG changes, excluding IFG-NFG and IFG-T2DM, conferred a higher risk for cancer mortality among women; using the IFG-ADA, the HRs ranged from 1.30 (1.02–1.66) for IFG-IFG to 2.90 (1.52–5.51) for NFG to T2DM category. However, using the IFG-WHO criteria, no significant association was found.

The results of women to men RHRs for CV and cancer mortality are shown in Figs. 3 and 4. Although the results were insignificant, women showed a higher risk for CV and cancer mortality than men. The multivariable-adjusted HRs of all studied covariates are presented in the Additional file 1: Tables S3–S5.

### Sensitivity analysis

To examine the robustness of our findings, we performed a series of sensitivity analyses. (1) To assess the impact of conversion to diabetes from the pre-diabetes status on our main analysis results, we excluded all T2DM cases as follows: First, we excluded T2DM from the first, second, and third examination (i.e., during about approximately six years from the recruitment time), second, we further excluded participants during the fourth examination (i.e., during about approximately nine years from the recruitment time) and repeated all analyses as shown in Additional file 1: Tables S6–S9. Accordingly, we generally found that incident IFG (using IFG-ADA) was still significantly associated with different mortality events, including all-cause, CV, and cancer mortality events in women. (2) To address the issue of reverse causality, we excluded participants who had early mortality events within a 3-year follow-up after the second examination, and the findings were generally in line with our main results (Additional file 1: Tables S10–S11**)**. In addition, the effect of sex differences in IFG-T2DM (using ADA definition) participants with respect to the incident CV mortality reached a significant level (1.99; 1.03–3.85). (3) We used physical activity as assessed by metabolic equivalent task (MET) as another covariate in the sensitivity analysis (among 13,978 out of 14,378 participants), considering the significant heterogeneity in assessing this confounder in different cohorts. Results did not show significant differences compared to our main findings (Additional file 1: Tables S12–S13**)**. (4) Finally, although the categorized FPG using predefined definitions were applied in the current study, we also examined the association between absolute change in FPG levels as a continuous variable with mortality outcomes (Additional file 1: Table S14). In women, risks of all-cause mortality, CV mortality, and cancer mortality increased by 9% (5–13%), 12% (6–19%), and 8% (1–14%) per 1-SD, respectively, and in men, only a significant risk was found for all-cause mortality events by 4% (0–8%, p = 0.04). Concerning gender differences, women had 8% (0–18%, p = 0.06) higher risk of CV mortality than men.

## Discussion

In the current study, using data from three large longitudinal cohorts, we examined the impact of three-year changes in FPG status on the incidence of all-cause, CV, and cancer mortality in more than 14,000 participants, without T2DM at baseline over two decades of follow-up. Our principal findings are as follows: (1) using the IFG-ADA, women who experienced incident IFG, persistent IFG, or progression from IFG to T2DM showed an increased risk of all-cause mortality; however, in men, the excess risk was observed only for those who converted to T2DM. Compared to men, women who remained in IFG status had a 24% higher risk for all-cause mortality. Using IFG-WHO, results were generally the same, except for incident IFG in women and IFG conversion to T2DM in men, which were no longer significant. (2) Regarding CV mortality, only among women, conversion to T2DM in IFG-ADA/WHO and NFG-WHO conferred increased risk. (3) For cancer mortality, based on IFG-ADA, NFG to IFG/T2DM and IFG to IFG categories were associated with a significantly greater risk in women only.

Concerning all-cause mortality, we found that IFG-ADA in different conditions, regardless of the conversion to T2DM, was a significant predictor for mortality events among women. In line with our findings, the Hoorn Study previously found that compared to individuals with baseline NFG, those who converted to T2DM during the six-year change from IFG-ADA and IFG-WHO had a higher risk of all-cause mortality in the age and sex-adjusted analysis [11]. However, in contrast to our findings, the researchers found that IFG status was associated with a higher risk of all-cause mortality only among those who converted to T2DM within a mean follow-up of 6.4 years.

Despite the irrefutable importance of hyperglycemia duration in developing diabetes-related macro- and microvascular complications [22], little is known about this issue in the pre-diabetes state. A retrospective cohort of the Korean National Health Insurance Service showed that the newly developed IFG-ADA was associated with an increased risk of all-cause mortality, mainly attributable to men and the elderly [8]. On the other hand, the current study also demonstrated that the newly developed IFG-ADA was significantly associated with all-cause and cancer mortality events among non-elderly women.

Regarding CV mortality, we observed more than a two-fold higher risk associated with the IFG-ADA conversion to T2DM, in the presence of well-known CVD risk factors, in women; when we used IFG-WHO, NFG conversion to T2DM also showed a significant risk. The Hoorn Study also found that conversion to T2DM from IFG-ADA/WHO was associated with more than a two-fold increased risk of CV mortality adjusted for age and sex [11]. A collaborative meta-analysis of 102 prospective studies found that the risk of coronary heart disease for T2DM was higher in women than in men, at 40–59 years than in the elderly population, and with fatal versus non-fatal disease [23]. What is more, Cai et al. [24] showed that pre-diabetes conferred an increased risk of composite CVD in the general population by 15%; however, no gender difference was found; additionally, the composite CVD risk associated with IFG was significantly higher in women with the prevalent atherosclerotic CVD than in men.

As another important finding, we demonstrated that women with persistent IFG during three years still had a higher risk for mortality events. By focusing on clinical trials, current evidence is not consistent or compelling regarding the effect of T2DM prevention on the risk of CVD or mortality [25]. Recently, the Diabetes Prevention Program Outcomes Study researchers found that neither metformin nor lifestyle intervention reduced major CVD events over 21 years despite long-term prevention of T2DM [26]. Hence, given the balance among benefits, risks, and costs of prevention, we speculate that prevention strategies might need to be considered earlier before subjects experience IFG, especially among women.

In this study, for the first time, we assessed the effects of the directions of FPG on the risk of cancer mortality. Generally, despite the absence of gender interaction, we found that women with IFG-ADA had a much higher risk of cancer mortality. Huang et al. [27], in a meta-analysis of 16 prospective studies, found a 15% increased risk of incidence and mortality of cancer associated with pre-diabetes; following subgroup analysis, men showed a higher risk than women; however, no gender interaction was identified. A meta-analysis of 19,239,302 people from 121 cohorts showed that T2DM was a risk factor for all-site cancer (incidence or fatal) in both sexes, with a significantly higher risk in women than in men by about 6% [28]. Findings from our study imply that even a single exposure to IFG-ADA puts women at a higher risk of cancer mortality, even after returning to normal FPG.

Comparing the impact of changing for IFG threshold from ADA to WHO in this pooled cohort study, we found that this alteration led to attenuation in the risk of different categories of FGP status in both genders; in women, NFG-IFG category for all-cause mortality and NFG-IFG and persistent IFG for cancer mortality did not remain as significant predictors. Moreover, the category of IFG-T2DM lost its significant association with all-cause mortality among men. Despite this, there was a significant overlap in the confidence intervals of HR for the mentioned FPG categories using different criteria. Therefore, it is impossible to conclude the superiority of ADA to WGO criteria firmly. In a meta-analysis including more than 10 million participants, researchers also did not find any differences between IFG-ADA and IFG-WHO for the risk of CV and all-cause mortality events using snapshot measurement of FGP level [2].

Current literature shows signals of sex differences for the outcomes in subjects with abnormal glucose levels [29–31]. Wang et al. [32], in a systematic review and meta-analysis, showed that compared to men with the same condition, women with T2DM had about 60% and 13% greater risk of coronary heart disease and all-cause mortality, respectively, although there was significant heterogeneity between studies; however, no such difference was observed for cancer mortality. The underlying reasons for these differences over the aging lifespan are less well-understood; differences in traditional CV risk factors do not explain the higher risk of CV mortality associated with T2DM in women compared to men [33]. Recently, we found that compared to men, women had greater exposure and burden of multiple risk factors before the onset of T2DM; the issue contributes to a higher CVD risk of T2DM among women. In the current study, we extend the previous research by showing that being in IFG status was associated with more than 20% higher risk for all-cause mortality among women compared to men.

## Strengths and limitations

Our study has several important strengths. First, a longitudinal population-based design over two decades of follow-up, with a large population size using data from three established cohorts, provided a diverse study population to improve generalizability. Second, including a substantial number of individuals without T2DM and CVD allowed us to analyze associations between all categories of changes in FPG status and risk of all-cause mortality separately in men and women. Third, careful adjustment for well-known CVD risk factors was performed in our data analysis. Finally, for the first time, we highlighted sex difference in the impact of FPG change on the risk of all-cause and cause-specific mortality events.

The results of our study need to be interpreted in the context of several limitations. First, the glycated hemoglobin A1c (HbA1c) and 2-h post-load glucose (2 h-PG) data were unavailable for all three cohorts, which may have led to the misclassification of T2DM and normoglycemia among participants. Second, study participants with IFG may have developed T2DM during the follow-up after the three years of examination. Hence, we did not exclude the possibility that the observed risks might be attributable to the development of T2DM during longer follow-ups. Third, although we adjusted our analyses for a wide variety of known covariates as appropriate for each outcome, residual confounding inherent in the observational studies, such as nutritional status and socioeconomic status, may alter the results. Fourth, we did not have enough events in the NFG to T2DM category for CV and cancer mortality events resulting in unstable effect sizes with wide confidence intervals. Fifth, data for cancer mortality analysis was only available for two cohorts, ARIC and TLGS. And last but not least, considering the age span of the study population, our findings might not be extrapolated to individuals > 60 years of age.

## Conclusion

In summary, among over 14,000 individuals free of T2DM and prevalent CVD, we examined the association between an approximately three-year change in IFG status with mortality events, with apparent sex differences in this pathway. Among women, the IFG status, whether as persistent, incident, or progression to T2DM, had a significant risk for all-cause mortality events in the presence of CVD risk factors. Moreover, the unfavorable impact of IFG was seen even among women who did not convert to T2DM status. However, in men, the excess risk was observed only for those who converted to T2DM. Additional work is needed to validate our findings and provide further information on the sexual dimorphism in the effects of glycemic directions on mortality events, the issue emphasized in precision medicine.

## Supplementary Information

**Additional file 1. Figure S1.** Study Flowchart. **Figures S2–S4.** Kaplan-Meier curves for mortality events stratified by six categories of FPG changes, separately in men and women. **Table S1.** Baseline characteristics of subjects stratified by respondents and non-respondents. **Table S2.** Baseline characteristics of subjects, stratified by cohort studies. **Tables S3–S5.** Multivariable adjusted HRs of confounders for the incidence of mortality events. **Tables S6–S7.** Multivariable adjusted HRs for the incidence of mortality events associated with different categories of fasting plasma glucose status changes after excluding participants with T2DM during visit 1 to visit 3. **Tables S8–S9.** Multivariable adjusted HRs for the incidence of mortality events associated with different categories of fasting plasma glucose status changes after excluding participants with T2DM during visit 1 to visit 4. **Tables S10–S11.**. Multivariable adjusted HRs for the incidence of mortality events associated with different categories of fasting plasma glucose status changes after excluding participants who had death events within a 3-year follow-up. **Tables S12–S13.** Multivariable adjusted HRs for the incidence of mortality events associated with different categories of fasting plasma glucose changes after further adjustment for physical activity covariate. **Table S14.** Multivariable adjusted HRs of 1-SD absolute changes in fasting plasma glucose with respect to mortality events.

## Data Availability

The datasets analyzed during the current study are available from the corresponding author on reasonable request.

## Abbreviations

FPG: Fasting plasma plucose
NFG: Normal fasting plucose
IFG: Impaired fasting glucose
IGT: Impaired glucose tolerance
T2DM: Type 2 diabetes
CV: Cardiovascular
CVD: Cardiovascular disease
ARIC: Atherosclerosis risk in communities study description
MESA: Multi-Ethnic Study of Atherosclerosis
TLGS: Tehran Lipid and Glucose Study
ADA: American Diabetes Association
WHO: World Health Organization
BMI: Body mass index
SBP: Systolic blood pressure
DBP: Diastolic blood pressure
TC: Total cholesterol
SD: Standard deviation
HR: Hazard ratio
RHR: Rario of hazard rario
CI: Confidence interval
