# Supplementary material for: Changes in Fasting plasma glucose status and risk of mortality events in individuals without diabetes over two decades of Follow-up: a pooled cohort analysis
Source: Cardiovasc Diabetol. 2022 Dec 3;21:267. doi: 10.1186/s12933-022-01709-z (PMC9719235; doi:10.1186/s12933-022-01709-z)
Supplement: Supplementary file 1 — Additional file 1. Figure S1. Study Flowchart. Figures S2–S4. Kaplan-Meier curves for mortality events stratified by six categories of FPG changes, separately in men and women. Table S1. Baseline characteristics of subjects stratified by respondents and non-respondents. Table S2. Baseline characteristics of subjects, stratified by cohort studies. Tables S3–S5. Multivariable adjusted HRs of confounders for the incidence of mortality events. Tables S6–S7. Multivariable adjusted HRs for the incidence of mortality events associated with different categories of fasting plasma glucose status changes after excluding participants with T2DM during visit 1 to visit 3. Tables S8–S9. Multivariable adjusted HRs for the incidence of mortality events associated with different categories of fasting plasma glucose status changes after excluding participants with T2DM during visit 1 to visit 4. Tables S10–S11.. Multivariable adjusted HRs for the incidence of mortality events associated with different categories of fasting plasma glucose status changes after excluding participants who had death events within a 3-year follow-up. Tables S12–S13. Multivariable adjusted HRs for the incidence of mortality events associated with different categories of fasting plasma glucose changes after further adjustment for physical activity covariate. Table S14. Multivariable adjusted HRs of 1-SD absolute changes in fasting plasma glucose with respect to mortality events. [file 12933_2022_1709_MOESM1_ESM.docx]

Study objective and design of cohorts

Atherosclerosis Risk in Communities Study (ARIC): ARIC aims to investigate the causes of atherosclerosis and its clinical outcomes, changes in cardiovascular risk factors, health care, and disease by race, gender, location, and date. The Cohort Component of ARIC began in 1987, and each of the four ARIC field centers in the United States, including Washington County, MD; Forsyth County, NC; Jackson, MS; and Minneapolis, MN, randomly selected and recruited a cohort sample of approximately 4,000 people aged 45–64 from a defined population in their community. A total of 15,792 participants received an extensive examination, including medical, social, and demographic data. Participants were reexamined every three-year, with the first screening (baseline) occurring in 1987–89, the second in 1990–92, the third in 1993–95, the fourth in 1996–98, and the fifth time in 2011–2013 (1). (More details are available at <https://sites.cscc.unc.edu/aric/description>)

Multi-Ethnic Study of Atherosclerosis (MESA): The MESA is a study of the characteristics of subclinical cardiovascular disease (disease detected non-invasively before it has produced clinical signs and symptoms) and the risk factors that predict progression to clinically overt cardiovascular disease or progression of the subclinical disease and it was initiated in 2000. MESA researchers study a diverse, population-based sample of 6,814 asymptomatic men and women aged 45–84 from six field centers across the United States, including Baltimore, MD (Johns Hopkins University); Chicago, IL (Northwestern University); Forsyth County, NC (Wake Forest University); Los Angeles County, CA (the University of California at Los Angeles); Northern Manhattan and Southern Bronx, NY (Columbia University); and St Paul, MN (University of Minnesota). Approximately 38 percent of the recruited participants were white, 28 percent African American, 22 percent Hispanic, and 12 percent Asian, predominantly of Chinese descent. The first examination, which began in July 2000 and was conducted over 24 months, was designed to be the most comprehensive. Six exams have been completed since 2000. Participants were contacted every 9 to 12 months throughout the study to assess clinical morbidity and mortality. The final 18 months of the study were dedicated to closing out and data analysis and publication (2). (More details are available at https://www.mesa-nhlbi.org/aboutMESA.aspx)

Tehran Lipid and Glucose Study (TLGS): The TLGS is a population-based longitudinal study conducted on individuals aged ≥3 years living in the urban area of Tehran, the capital of Iran. This study aimed to determine the prevalence and incidence of non-communicable diseases and their related risk factors. It also looked at developing a healthy lifestyle to counteract these risk factors. TLGS enrollment was carried out in two phases, the first from January 31, 1999, to July 03, 2001, with a second enrollment phase from October 20, 2001, until September 22, 2005. Data collection is planned to continue for at least 20 years with approximately 3-year intervals (i.e., phase III: 2005–2008, phase IV: 2008–2011, phase V: 2011–2014, and phase VI: 2015–2018) (3).

| Table S1. Baseline characteristics of subjects stratified by respondents and non-respondents. | | | | | |
| --- | --- | --- | --- | --- | --- |
| Variables | All  (n=17836) | Non-respondent  (n=3458) | Respondent  (n=14378) | 95% CI |  |
| Continuous variables, mean ± SD |  | | | |  |
| Age (years) | 49.6 ± 7.2 | 48.1 ± 8 | 50 ± 6.9 | -2.10 – -1.52 |  |
| SBP (mmHg) | 117.5 ± 17.4 | 119 ± 19.8 | 117.1 ± 16.8 | 1.16– 2.61 |  |
| DBP (mmHg) | 73.8 ± 10.8 | 75 ± 11.9 | 73.6 ± 10.5 | 1.03 – 1.91 |  |
| BMI (kg/m^2^) | 27.7 ± 5.2 | 27.9 ± 5.5 | 27.6 ± 5.1 | 0.01 – 0.44 |  |
| TC (mmol/L) | 5.3 ± 1.1 | 5.3 ± 1.1 | 5.3 ± 1.0 | -0.08 – 0.01 |  |
| FPG (mmol/L) | 5.3 ± 0.6 | 5.3 ± 0.6 | 5.3 ± 0.6 | -0.01 – 0.04 |  |
| Categorical variables, number (%) |  | | | |  |
| Women | 10184 (57.1) | 1912 (55.3) | 8272 (57.5) | -0.02 – 0.00 |  |
| Ever smoker | 8351 (46.8) | 1669 (48.3) | 6682 (46.5) | 0.01 – 0.03 |  |
| Hypertension (yes) | 4306 (24.1) | 908 (26.3) | 3398 (23.6) | 0.02 – 0.06 |  |
| Hypercholesterolemia (yes) | 9256 (51.9) | 1606 (46.4) | 7650 (53.2) | -0.02 – 0.01 |  |
| As appropriate, data are shown as mean ± standard deviation (SD) or number (percent).  SBP: systolic blood pressure; DBP: diastolic blood pressure; BMI: body mass index; FPG: fasting plasma glucose; TC: total cholesterol. | | | | | |

| Table S2. Baseline characteristics of subjects, stratified by cohort studies. | | | | |  |
| --- | --- | --- | --- | --- | --- |
| Variables | All  (n=14378) | ARIC  (n=8351) | MESA  (n=2555) | TLGS  (n=3472) | |
| Continuous variables, mean ± SD |  |  | | | |
| Age (years) | 50.0 ± 6.9 | 52.1 ± 4.5 | 52.4 ± 4.5 | 43 ± 8.3 | |
| SBP (mmHg) | 117.1 ± 16.8 | 118 ± 16.9 | 118.2 ± 18.0 | 114.2 ± 15.4 | |
| DBP (mmHg) | 73.6 ± 10.5 | 73.2 ± 10.7 | 72.2 ± 10.3 | 75.3 ± 10.1 | |
| BMI (kg/m^2^) | 27.6 ± 5.1 | 27.2 ± 5.1 | 28.4 ± 5.5 | 28.1 ± 4.5 | |
| TC (mmol/L) | 5.3 ± 1.0 | 5.5 ± 1.0 | 5.1 ± 0.9 | 5.1 ± 1.0 | |
| FPG (mmol/L) | 5.3 ± 0.6 | 5.5 ± 0.5 | 4.9 ± 0.6 | 5.1 ± 0.5 | |
| Categorical variables, number (%) |  |  | | | |
| Women | 8272 (57.5) | 4847 (58.0) | 1368 (53.5) | 2057 (59.2) | |
| Ever smoker | 6682 (46.5) | 4685 (56.1) | 1243 (48.6) | 754 (21.7) | |
| Hypertension (yes) | 3398 (23.6) | 2197 (26.3) | 750 (29.4) | 451 (13.0) | |
| Hypercholesterolemia (yes) | 7650 (53.2) | 4980 (59.6) | 1206 (47.2) | 1464 (42.2) | |
| As appropriate, data are shown as mean ± standard deviation (SD) or number (percent).  ARIC: Atherosclerosis Risk in Communities; MESA: Multi-Ethnic Study of Atherosclerosis; TLGS: Tehran Lipid and Glucose Study; SBP: systolic blood pressure; DBP: diastolic blood pressure; BMI: body mass index; FPG: fasting plasma glucose; TC: total cholesterol | | | | |  |

| Table S3. Multivariable adjusted HRs (CIs 95%) of confounders for the incidence of all-cause mortality | | | | |
| --- | --- | --- | --- | --- |
|  | HRs of confounders in the model with IFG-ADA | | HRs of confounders in the model with IFG-WHO | |
| Variables | **HR (95% CI)** | **p-value** | **HR (95% CI)** | **p-value** |
| Age (years) | 1.10 (1.09–1.11) | <0.001 | 1.10 (1.09–1.11) | <0.001 |
| BMI (kg/m^2^) | 1.01 (1.00–1.02) | 0.011 | 1.01 (1.00–1.02) | 0.008 |
| BMI-Change (kg/m^2^) | 0.97 (0.95–0.99) | 0.010 | 0.97 (0.95–0.99) | 0.013 |
| Hypertension | 1.45 (1.33–1.59) | <0.001 | 1.45 (1.33–1.59) | <0.001 |
| Hypercholesterolemia | 0.94 (0.87–1.03) | 0.181 | 0.94 (0.87–1.02) | 0.164 |
| Smoking | 1.79 (1.64–1.96) | <0.001 | 1.79 (1.63–1.95) | <0.001 |
| HR: hazard ratio; CI: confidence interval; BMI: body mass index; ADA: American Diabetes Association; WHO: World Health Organization | | | | |

| Table S4. Multivariable adjusted HRs (CIs 95%) of confounders for the incidence of CV mortality | | | | |
| --- | --- | --- | --- | --- |
|  | HRs of confounders in the model with IFG-ADA | | HRs of confounders in the model with IFG-WHO | |
| Variables | **HR (95% CI)** | **p-value** | **HR (95% CI)** | **p-value** |
| Age (years) | 1.09 (1.07–1.11) | <0.001 | 1.09 (1.07–1.11) | <0.001 |
| BMI (kg/m^2^) | 1.03 (1.01–1.05) | <0.001 | 1.03 (1.01–1.05) | <0.001 |
| BMI-Change (kg/m^2^) | 0.97 (0.92–1.01) | 0.122 | 0.97 (0.92–1.01) | 0.137 |
| Hypertension | 2.21 (1.87–2.62) | <0.001 | 2.21 (1.87–2.62) | <0.001 |
| Hypercholesterolemia | 1.10 (0.93–1.31) | 0.242 | 1.10 (0.93–1.30) | 0.249 |
| Smoking | 1.61 (1.35–1.91) | <0.001 | 1.61 (1.35–1.91) | <0.001 |
| HR: hazard ratio; CI: confidence interval; CV: cardiovascular; BMI: body mass index; ADA: American Diabetes Association; WHO: World Health Organization | | | | |

| Table S5. Multivariable adjusted HRs (CIs 95%) of confounders for the incidence of cancer mortality | | | | |
| --- | --- | --- | --- | --- |
|  | HRs of confounders in the model with IFG-ADA | | HRs of confounders in the model with IFG-WHO | |
| Variables | **HR (95% CI)** | **p-value** | **HR (95% CI)** | **p-value** |
| Age (years) | 1.08 (1.07 – 1.10) | <0.001 | 1.08 (1.07 – 1.10) | 0.032 |
| BMI (kg/m^2^) | 1.00 (0.99 – 1.02) | 0.698 | 1.00 (0.99 – 1.02) | 0.528 |
| BMI-Change (kg/m^2^) | 0.96 (0.91 – 0.99) | 0.027 | 0.96 (0.92 – 1.00) | 0.047 |
| Hypertension | 1.12 (0.96 – 1.30) | 0.145 | 1.12 (0.96 – 1.30) | 0.151 |
| Hypercholesterolemia | 0.91 (0.79 – 1.04) | 0.158 | 0.91 (0.79 – 1.04) | 0.159 |
| Smoking | 2.04 (1.77 – 2.36) | <0.001 | 2.04 (1.76 – 2.35) | <0.001 |
| HR: hazard ratio; CI: confidence interval; BMI: body mass index; ADA: American Diabetes Association; WHO: World Health Organization | | | | |

| Table S6. Multivariable adjusted HRs (CIs 95%) for the incidence of mortality events associated with different categories of fasting plasma glucose status changes (based on ADA definition) after excluding participants with T2DM during visit 1 to visit 3. (n=11985) | | | | |
| --- | --- | --- | --- | --- |
| Outcome |  | Women | Men | Women to Men RHRs |
| All-cause mortality |  |  |  |  |
|  | NFG to NFG | Reference | Reference | Reference |
|  | NFG to IFG | **1.28 (1.07-1.52)** | 1.05 (0.88-1.26) | 1.21 (0.95-155) |
|  | IFG to NFG | 1.24 (0.96-1.62) | 0.96 (0.73-1.25) | 1.30 (0.89-1.89) |
|  | IFG to IFG | 1.08 (0.90-1.30) | 0.91 (0.76-1.08) | 1.19 (0.93-1.53) |
| CV Mortality |  |  |  |  |
|  | NFG to NFG | Reference | Reference | Reference |
|  | NFG to IFG | **1.43 (1.02-1.99)** | 1.02 (0.72-1.43) | 1.40 (0.87-2.25) |
|  | IFG to NFG | 1.12 (0.65-1.90) | 0.86 (0.51-1.47) | 1.29 (0.61-2.73) |
|  | IFG to IFG | 0.98 (0.68-1.42) | 0.85 (0.61-1.18) | 1.16 (0.71-1.88) |
| Cancer mortality* |  |  |  |  |
|  | NFG to NFG | Reference | Reference | Reference |
|  | NFG to IFG | **1.43 (1.09-1.87)** | 1.23 (0.91-1.67) | 1.15 (0.77-1.73) |
|  | IFG to NFG | **1.53 (1.03-2.26)** | 1.13 (0.72-1.77) | 1.35 (0.74-2.44) |
|  | IFG to IFG | 1.21 (0.91-1.62) | 1.11 (0.83-1.49) | 1.09 (0.73-1.64) |
| HR: hazard ratio; CI: confidence interval; RHR: ratios of HR; ADA: American Diabetes Association; NFG: normal fasting glucose; IFG: impaired fasting glucose based on ADA cut-offs (100 mg/dl); CV: cardiovascular; T2DM: type 2 diabetes mellitus. All models were adjusted for age, BMI, BMI-Change, smoking status, hypertension, and hypercholesterolemia. Significant values are bold.  *Cancer mortality was assessed only among participants from ARIC and TLGS studies. (n=9683) | | | | |

| Table S7. Multivariable adjusted HRs (CIs 95%) for the incidence of mortality events associated with different categories of fasting plasma glucose status changes (based on WHO definition) after excluding participants with T2DM during visit 1 to visit 3. (n=11985) | | | | |
| --- | --- | --- | --- | --- |
| Outcome |  | Women | Men | Women to Men RHRs |
| All-cause mortality |  |  |  |  |
|  | NFG to NFG | Reference | Reference | Reference |
|  | NFG to IFG | 1.12 (0.90-1.39) | 0.96 (0.79-1.16) | 1.17 (0.88-1.56) |
|  | IFG to NFG | 1.16 (0.78-1.72) | 0.91 (0.65-1.28) | 1.27 (0.75-2.13) |
|  | IFG to IFG | 1.31 (0.94-1.81) | 0.99 (0.76-1.30) | 1.32 (0.87-2.00) |
| CV Mortality |  |  |  |  |
|  | NFG to NFG | Reference | Reference | Reference |
|  | NFG to IFG | 1.12 (0.75-1.68) | 0.92 (0.64-1.32) | 1.22 (0.71-2.08) |
|  | IFG to NFG | 0.74 (0.30-1.80) | 0.89 (0.47-1.68) | 0.83 (0.28-2.49) |
|  | IFG to IFG | 1.30 (0.72-2.35) | 0.92 (0.55-1.52) | 1.41 (0.65-3.06) |
| Cancer mortality* |  |  |  |  |
|  | NFG to NFG | Reference | Reference | Reference |
|  | NFG to IFG | 1.21 (0.86-1.70) | 0.92 (0.66-1.26) | 1.32 (0.83-2.10) |
|  | IFG to NFG | **1.83 (1.08-3.09)** | 0.94 (0.54-1.64) | 1.95 (0.91-4.20) |
|  | IFG to IFG | 1.36 (0.80-2.30) | 1.15 (0.75-1.76) | 1.18 (0.60-2.31) |
| HR: hazard ratio; CI: confidence interval; RHR: ratios of HR; WHO: World Health Organization; NFG: normal fasting glucose; IFG: impaired fasting glucose based on WHO cut-offs (110 mg/dl); CV: cardiovascular; T2DM: type 2 diabetes mellitus. All models were adjusted for age, BMI, BMI-Change, smoking status, hypertension, and hypercholesterolemia. Significant values are bold.  *Cancer mortality was assessed only among participants from ARIC and TLGS studies. (n=9683) | | | | |

| Table S8. Multivariable adjusted HRs (CIs 95%) for the incidence of mortality events associated with different categories of fasting plasma glucose status changes (based on ADA definition) after excluding participants with T2DM during visit 1 to visit 4. (n=10558) | | | | |
| --- | --- | --- | --- | --- |
| Outcome |  | Women | Men | Women to Men RHRs |
| All-cause mortality |  |  |  |  |
|  | NFG to NFG | Reference | Reference | Reference |
|  | NFG to IFG | **1.30 (1.07-1.58)** | 1.09 (0.89-1.34) | 1.19 (0.90-1.58) |
|  | IFG to NFG | 1.18 (0.87-1.60) | 1.02 (0.76-1.38) | 1.16 (0.76-1.77) |
|  | IFG to IFG | 1.10 (0.89-1.36) | 0.91 (0.75-1.11) | 1.20 (0.90-1.60) |
| CV Mortality |  |  |  |  |
|  | NFG to NFG | Reference | Reference | Reference |
|  | NFG to IFG | **1.49 (1.02-2.17)** | 1.09 (0.72-1.64) | 1.37 (0.79-2.37) |
|  | NFG to T2DM | 1.05 (0.57-1.94) | 1.19 (0.68-2.08) | 0.88 (0.39-2.02) |
|  | IFG to IFG | 0.92 (0.60-1.43) | 0.89 (0.60-1.32) | 1.04 (0.58-1.85) |
| Cancer mortality* |  |  |  |  |
|  | NFG to NFG | Reference | Reference | Reference |
|  | NFG to IFG | **1.40 (1.03-1.92)** | 1.30 (0.92-1.83) | 1.08 (0.68-1.71) |
|  | IFG to NFG | 1.47 (0.93-2.32) | 1.12 (0.67-1.86) | 1.31 (0.66-2.60) |
|  | IFG to IFG | 1.32 (0.95-1.84) | 1.19 (0.86-1.66) | 1.11 (0.70-1.75) |
| HR: hazard ratio; CI: confidence interval; RHR: ratios of HR; ADA: American Diabetes Association; NFG: normal fasting glucose; IFG: impaired fasting glucose based on ADA cut-offs (100 mg/dl); CV: cardiovascular; T2DM: type 2 diabetes mellitus. All models were adjusted for age, BMI, BMI-Change, smoking status, hypertension, and hypercholesterolemia. Significant values are bold.  *Cancer mortality was assessed only among participants from ARIC and TLGS studies. (n=8433) | | | | |

| Table S9. Multivariable adjusted HRs (CIs 95%) for the incidence of mortality events associated with different categories of fasting plasma glucose status changes (based on ADA definition) after excluding participants with T2DM during visit 1 to visit 4. (n=10558) | | | | |
| --- | --- | --- | --- | --- |
| Outcome |  | Women | Men | Women to Men RHRs |
| All-cause mortality |  |  |  |  |
|  | NFG to NFG | Reference | Reference | Reference |
|  | NFG to IFG | 1.10 (0.85-1.42) | 0.93 (0.74-1.17) | 1.18 (0.84-1.65) |
|  | IFG to NFG | 1.13 (0.70-1.81) | 0.90 (0.62-1.32) | 1.25 (0.68-2.29) |
|  | IFG to IFG | 1.26 (0.85-1.86) | 1.00 (0.72-1.37) | 1.26 (0.76-2.09) |
| CV Mortality |  |  |  |  |
|  | NFG to NFG | Reference | Reference | Reference |
|  | NFG to IFG | 0.98 (0.59-1.64) | 0.93 (0.60-1.44) | 1.06 (0.54-2.07) |
|  | IFG to NFG | 1.06 (0.43-2.60) | 1.13 (0.57-2.22) | 0.94 (0.31-2.90) |
|  | IFG to IFG | 1.25 (0.61-2.57) | 1.15 (0.65-2.06) | 1.08 (0.43-2.71) |
| Cancer mortality* |  |  |  |  |
|  | NFG to NFG | Reference | Reference | Reference |
|  | NFG to IFG | 1.17 (0.78-1.74) | 0.81 (0.55-1.19) | 1.44 (0.83-2.51) |
|  | IFG to NFG | 1.52 (0.78-2.97) | 0.96 (0.52-1.76) | 1.59 (0.64-3.93) |
|  | IFG to IFG | 1.39 (0.76-2.57) | 1.23 (0.75-2.01) | 1.13 (0.52-2.47) |
| HR: hazard ratio; CI: confidence interval; RHR: ratios of HR; WHO: World Health Organization; NFG: normal fasting glucose; IFG: impaired fasting glucose based on WHO cut-offs (110 mg/dl); CV: cardiovascular; T2DM: type 2 diabetes mellitus. All models were adjusted for age, BMI, BMI-Change, smoking status, hypertension, and hypercholesterolemia.  *Cancer mortality was assessed only among participants from ARIC and TLGS studies. (n=8433) | | | | |

| Table S10. Multivariable adjusted HRs (CIs 95%) for the incidence of mortality events associated with different categories of fasting plasma glucose status changes (based on ADA definition) after excluding participants who had death events within 3-year follow-up (after the second examination). (n=14248) | | | | |
| --- | --- | --- | --- | --- |
| Outcome |  | Women | Men | Women to Men RHRs |
| All-cause mortality |  |  |  |  |
|  | NFG to NFG | Reference | Reference | Reference |
|  | NFG to IFG | **1.29 (1.10-1.51)** | 1.09 (0.92-1.28) | 1.18 (0.95-1.48) |
|  | NFG to T2DM | **2.02 (1.27-3.20)** | **1.91 (1.24-2.95)** | 1.05 (0.56-1.98) |
|  | IFG to NFG | 1.26 (0.99-1.59) | 1.04 (0.82-1.32) | 1.21 (0.87-1.69) |
|  | IFG to IFG | 1.16 (0.99-1.36) | 0.95 (0.81-1.10) | 1.23 (0.99-1.53) |
|  | IFG to T2DM | **1.63 (1.24-2.16)** | 1.27 (0.98-1.65) | 1.29 (0.88-1.87) |
| CV Mortality |  |  |  |  |
|  | NFG to NFG | Reference | Reference | Reference |
|  | NFG to IFG | 1.30 (0.95-1.78) | 1.04 (0.76-1.42) | 1.26 (0.81-1.95) |
|  | NFG to T2DM | 1.39 (0.51-3.79) | 1.56 (0.68-3.59) | 0.89 (0.24-3.25) |
|  | IFG to NFG | 1.12 (0.69-1.81) | 0.90 (0.56-1.46) | 1.23 (0.63-2.42) |
|  | IFG to IFG | 1.10 (0.80-1.51) | 0.87 (0.65-1.17) | 1.26 (0.83-1.93) |
|  | IFG to T2DM | **2.26 (1.45-3.53)** | 1.13 (0.69-1.88) | **1.99 (1.03-3.85)** |
| Cancer mortality* |  |  |  |  |
|  | NFG to NFG | Reference | Reference | Reference |
|  | NFG to IFG | **1.43 (1.13-1.83)** | 1.26 (0.96-1.65) | 1.14 (0.79-1.64) |
|  | NFG to T2DM | **2.71 (1.38-5.34)** | 1.46 (0.59-3.58) | 1.86 (0.61-5.71) |
|  | IFG to NFG | 1.36 (0.94-1.96) | 1.27 (0.86-1.87) | 1.07 (0.63-1.82) |
|  | IFG to IFG | 1.25 (0.98-1.61) | 1.09 (0.84-1.42) | 1.15 (0.80-1.64) |
|  | IFG to T2DM | 1.43 (0.88-2.32) | 1.31 (0.82-2.08) | 1.09 (0.56-2.12) |
| HR: hazard ratio; CI: confidence interval; RHR: ratios of HR; ADA: American Diabetes Association; NFG: normal fasting glucose; IFG: impaired fasting glucose based on ADA cut-offs (100 mg/dl); CV: cardiovascular; T2DM: type 2 diabetes mellitus. All models were adjusted for age, BMI, BMI-Change, smoking status, hypertension, and hypercholesterolemia. Significant values are bold.  *Cancer mortality was assessed only among participants from ARIC and TLGS studies. (n=11710) | | | | |

| Table S11. Multivariable adjusted HRs (CIs 95%) for the incidence of mortality events associated with different categories of fasting plasma glucose changes (based on WHO definition) after excluding participants who had death events within 3-year follow-up (after the second examination). (n=14248) | | | | |
| --- | --- | --- | --- | --- |
| Outcome |  | Women | Men | Women to Men RHRs |
| All-cause mortality |  |  |  |  |
|  | NFG to NFG | Reference | Reference | Reference |
|  | NFG to IFG | 1.19 (0.84-1.69) | 1.01 (0.75-1.37) | 1.20 (0.94-1.53) |
|  | NFG to T2DM | **2.14 (1.23-3.71)** | 1.39 (0.73-2.64) | 1.18 (0.75-1.87) |
|  | IFG to NFG | 0.81 (0.38-1.72) | 0.82 (0.46-1.47) | 1.26 (0.80-1.99) |
|  | IFG to IFG | 1.40 (0.86-2.28) | 0.87 (0.56-1.34) | 1.37 (0.97-1.92) |
|  | IFG to T2DM | **1.85 (1.10-3.12)** | 1.17 (0.69-1.99) | 1.12 (0.73-1.72) |
| CV Mortality |  |  |  |  |
|  | NFG to NFG | Reference | Reference | Reference |
|  | NFG to IFG | 1.19 (0.84-1.69) | 1.01 (0.75-1.37) | 1.18 (0.74-1.86) |
|  | NFG to T2DM | **2.14 (1.23-3.71)** | 1.39 (0.73-2.64) | 1.54 (0.67-3.56) |
|  | IFG to NFG | 0.81 (0.38-1.72) | 0.82 (0.46-1.47) | 0.99 (0.38-2.57) |
|  | IFG to IFG | 1.40 (0.86-2.28) | 0.87 (0.56-1.34) | 1.62 (0.85-3.10) |
|  | IFG to T2DM | **1.85 (1.10-3.12)** | 1.17 (0.69-1.99) | 1.58 (0.76-3.30) |
| Cancer mortality* |  |  |  |  |
|  | NFG to NFG | Reference | Reference | Reference |
|  | NFG to IFG | 1.26 (0.95-1.67) | 0.92 (0.70-1.22) | 1.36 (0.92-2.02) |
|  | NFG to T2DM | 1.58 (0.90-2.77) | 1.23 (0.65-2.32) | 1.29 (0.55-2.99) |
|  | IFG to NFG | 1.42 (0.86-2.36) | 0.91 (0.56-1.48) | 1.57 (0.78-3.16) |
|  | IFG to IFG | 1.36 (0.89-2.08) | 1.18 (0.83-1.66) | 1.15 (0.67-1.99) |
|  | IFG to T2DM | 1.46 (0.86-2.47) | 1.15 (0.70-1.89) | 1.27 (0.62-2.59) |
| HR: hazard ratio; CI: confidence interval; RHR: ratios of HR; WHO: World Health Organization; NFG: normal fasting glucose; IFG: impaired fasting glucose based on WHO cut-offs (110 mg/dl); CV: cardiovascular; T2DM: type 2 diabetes mellitus. All models were adjusted for age, BMI, BMI-Change, smoking status, hypertension, and hypercholesterolemia. Significant values are bold.  *Cancer mortality was assessed only among participants from ARIC and TLGS studies. (n=11710) | | | | |

| Table S12. Multivariable adjusted HRs (CIs 95%) for the incidence of mortality events associated with different categories of fasting plasma glucose changes (based on ADA definition) after further adjustment for physical activity covariate. (n=13978) | | | | |
| --- | --- | --- | --- | --- |
| Outcome |  | Women | Men | Women to Men RHRs |
| All-cause mortality |  |  |  |  |
|  | NFG to NFG | Reference | Reference | Reference |
|  | NFG to IFG | **1.29 (1.10-1.51)** | 1.11 (0.95-1.31) | 1.16 (0.93-1.45) |
|  | NFG to T2DM | **2.03 (1.29-3.19)** | **1.89 (1.21-2.95)** | 1.07 (0.57-2.01) |
|  | IFG to NFG | 1.22 (0.96-1.55) | 1.10 (0.87-1.40) | 1.11 (0.79-1.54) |
|  | IFG to IFG | 1.15 (0.98-1.35) | 0.96 (0.82-1.12) | 1.20 (0.96-1.49) |
|  | IFG to T2DM | **1.58 (1.20-2.09)** | 1.27 (0.98-1.66) | 1.24 (0.85-1.80) |
| CV Mortality |  |  |  |  |
|  | NFG to NFG | Reference | Reference | Reference |
|  | NFG to IFG | 1.24 (0.90-1.72) | 1.07 (0.78-1.46) | 1.16 (0.74-1.81) |
|  | NFG to T2DM | 1.41 (0.51-3.84) | 1.63 (0.71-3.76) | 0.86 (0.23-3.15) |
|  | IFG to NFG | 1.03 (0.62-1.70) | 1.01 (0.63-1.61) | 1.02 (0.51-2.02) |
|  | IFG to IFG | 1.08 (0.78-1.49) | 0.92 (0.68-1.23) | 1.17 (0.76-1.80) |
|  | IFG to T2DM | **2.24 (1.43-3.49)** | 1.36 (0.85-2.18) | 1.64 (0.87-3.08) |
| Cancer mortality* |  |  |  |  |
|  | NFG to NFG | Reference | Reference | Reference |
|  | NFG to IFG | **1.53 (1.21-1.93)** | 1.19 (0.92-1.55) | 1.28 (0.90-1.82) |
|  | NFG to T2DM | **2.86 (1.50-5.44)** | 1.09 (0.40-2.97) | 2.62 (0.80-8.55) |
|  | IFG to NFG | 1.40 (0.98-2.00) | 1.27 (0.88-1.84) | 1.10 (0.66-1.84) |
|  | IFG to IFG | **1.31 (1.02-1.67)** | 1.03 (0.80-1.33) | 1.27 (0.90-1.80) |
|  | IFG to T2DM | 1.38 (0.85-2.24) | 1.13 (0.71-1.81) | 1.22 (0.63-2.37) |
| HR: hazard ratio; CI: confidence interval; RHR: ratios of HR; ADA: American Diabetes Association; NFG: normal fasting glucose; IFG: impaired fasting glucose based on ADA cut-offs (100 mg/dl); CV: cardiovascular; T2DM: type 2 diabetes mellitus. All models were adjusted for age, BMI, BMI-Change, smoking status, hypertension, hypercholesterolemia, and physical activity. Significant values are bold.  *Cancer mortality was assessed only among participants from ARIC and TLGS studies. (n=11424) | | | | |

| Table S13. Multivariable adjusted HRs (CIs 95%) for the incidence of mortality events associated with different categories of fasting plasma glucose changes (based on WHO definition) after further adjustment for physical activity covariate. (n=13978) | | | | |
| --- | --- | --- | --- | --- |
| Outcome |  | Women | Men | Women to Men RHRs |
| All-cause mortality |  |  |  |  |
|  | NFG to NFG | Reference | Reference | Reference |
|  | NFG to IFG | 1.15 (0.95-1.38) | 0.97 (0.82-1.15) | 1.18 (0.92-1.51) |
|  | NFG to T2DM | **1.80 (1.31-2.47)** | **1.55 (1.11-2.17)** | 1.16 (0.73-1.83) |
|  | IFG to NFG | 1.14 (0.81-1.61) | 1.01 (0.76-1.34) | 1.13 (0.73-1.77) |
|  | IFG to IFG | **1.34 (1.02-1.74)** | 0.94 (0.75-1.18) | **1.42 (1.00-2.00)** |
|  | IFG to T2DM | 1.37 (0.99-1.90) | 1.22 (0.92-1.61) | 1.13 (0.74-1.73) |
| CV Mortality |  |  |  |  |
|  | NFG to NFG | Reference | Reference | Reference |
|  | NFG to IFG | 1.02 (0.70-1.49) | 0.99 (0.73-1.35) | 1.03 (0.64-1.68) |
|  | NFG to T2DM | **2.13 (1.23-3.69)** | 1.51 (0.82-2.78) | 1.41 (0.62-3.19) |
|  | IFG to NFG | 0.83 (0.39-1.77) | 0.96 (0.56-1.65) | 0.87 (0.34-2.20) |
|  | IFG to IFG | 1.42 (0.87-2.32) | 0.80 (0.52-1.25) | 1.77 (0.92-3.40) |
|  | IFG to T2DM | **1.84 (1.09-3.10)** | 1.33 (0.82-2.17) | 1.38 (0.68-2.79) |
| Cancer mortality* |  |  |  |  |
|  | NFG to NFG | Reference | Reference | Reference |
|  | NFG to IFG | 1.27 (0.96-1.67) | 0.89 (0.67-1.17) | 1.42 (0.97-2.10) |
|  | NFG to T2DM | 1.57 (0.91-2.69) | 1.04 (0.53-2.03) | 1.50 (0.64-3.53) |
|  | IFG to NFG | 1.35 (0.82-2.24) | 1.00 (0.63-1.57) | 1.35 (0.69-2.66) |
|  | IFG to IFG | 1.39 (0.92-2.10) | 1.03 (0.72-1.47) | 1.35 (0.79-2.31) |
|  | IFG to T2DM | 1.38 (0.81-2.33) | 1.00 (0.60-1.66) | 1.37 (0.67-2.83) |
| HR: hazard ratio; CI: confidence interval; RHR: ratios of HR; WHO: World Health Organisation; NFG: normal fasting glucose; IFG: impaired fasting glucose based on ADA cut-offs (110 mg/dl); CV: cardiovascular; T2DM: type 2 diabetes mellitus. All models were adjusted for age, BMI, BMI-Change, smoking status, hypertension, hypercholesterolemia, and physical activity. Significant values are bold.  *Cancer mortality was assessed only among participants from ARIC and TLGS studies. (n=11424) | | | | |

| Table S14. Multivariable adjusted HRs (CIs 95%) of 1-SD absolute changes in fasting plasma glucose with respect to mortality events | | | | |
| --- | --- | --- | --- | --- |
| Outcome | Model | Women | Men | Women to Men RHRs |
| All-cause mortality | 1 | **1.09 (1.05-1.13)** | **1.04 (1.00-1.08)** | 1.05 (0.99-1.10) |
|  | 2 | **1.09 (1.05-1.13)** | **1.04 (1.00-1.08)** | 1.04 (0.99-1.10) |
| CV Mortality | 1 | **1.12 (1.06-1.19)** | 1.04 (0.97-1.10) | **1.08 (1.00-1.17)** |
|  | 2 | **1.12 (1.06-1.19)** | 1.04 (0.97-1.10) | **1.08 (1.00-1.18)** |
| Cancer mortality | 1 | **1.08 (1.01-1.14)** | 0.99 (0.90-1.09) | 1.08 (0.97-1.21) |
|  | 2 | **1.07 (1.01-1.14)** | 1.00 (0.91-1.10) | 1.08 (0.96-1.20) |
| SD: standard deviation; HR: hazard ratio; RHR: ratios of HR; CI: confidence interval  Model 1 adjusted for age, BMI, BMI-Change, smoking status, hypertension, and hypercholesterolemia.  Model 2 further adjusted for baseline fasting plasma glucose. Significant values are bold. | | | | |


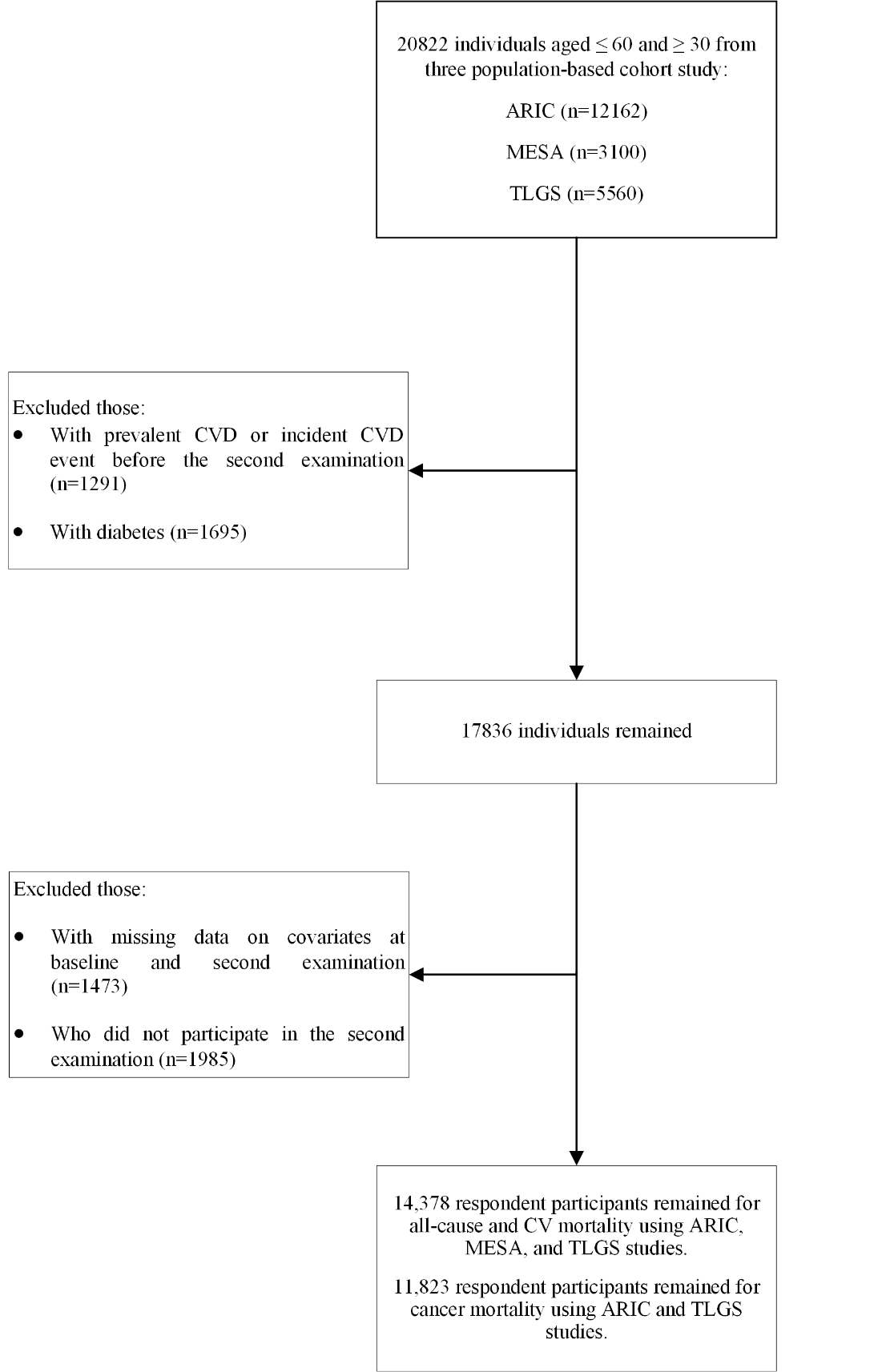


**Figure S1.** Study Flowchart

CV: Cardiovascular; ARIC: Atherosclerosis Risk in Communities; MESA: Multi-Ethnic Study of Atherosclerosis; TLGS: Tehran Lipid and Glucose Study


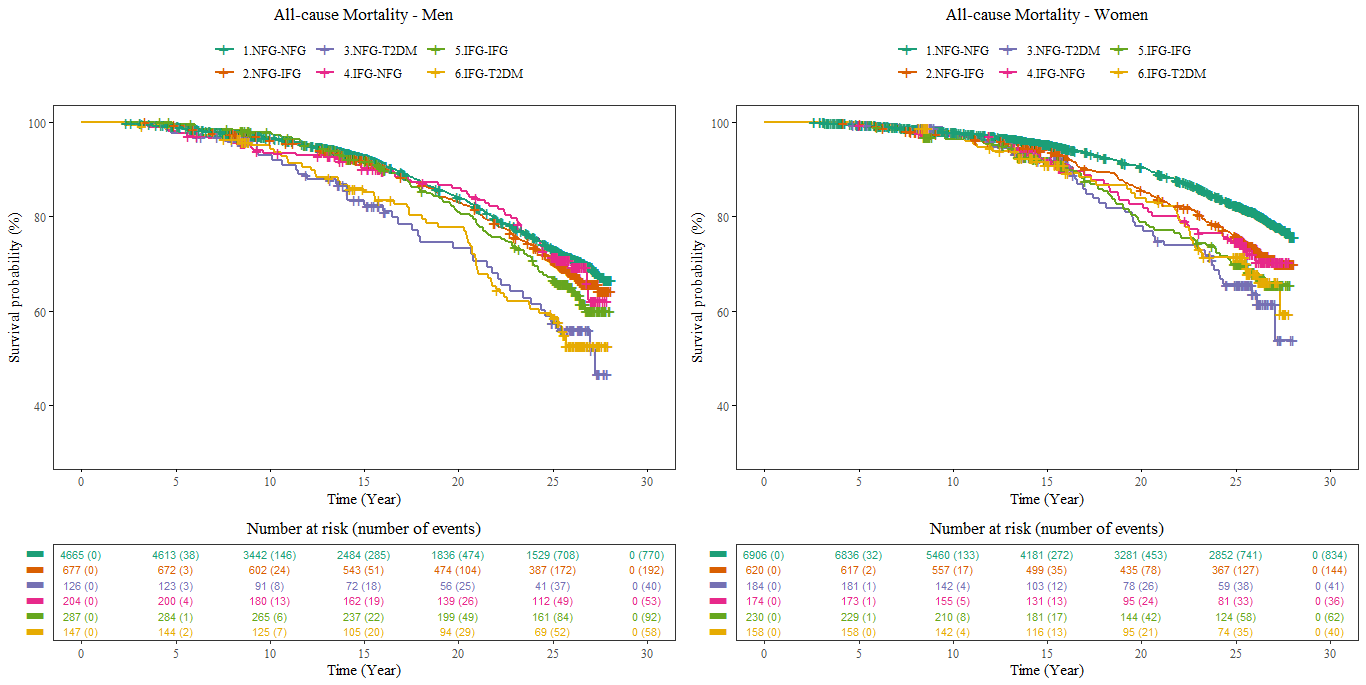


**Figure S2.** Kaplan-Meier curves for all-cause mortality stratified by six categories of FPG changes, separately in men and women.

FPG: fasting plasma glucose; NFG: normal fasting glucose; IFG: impaired fasting glucose (based on ADA cut-off (100 mg/dl)); T2DM: type 2 diabetes mellitus


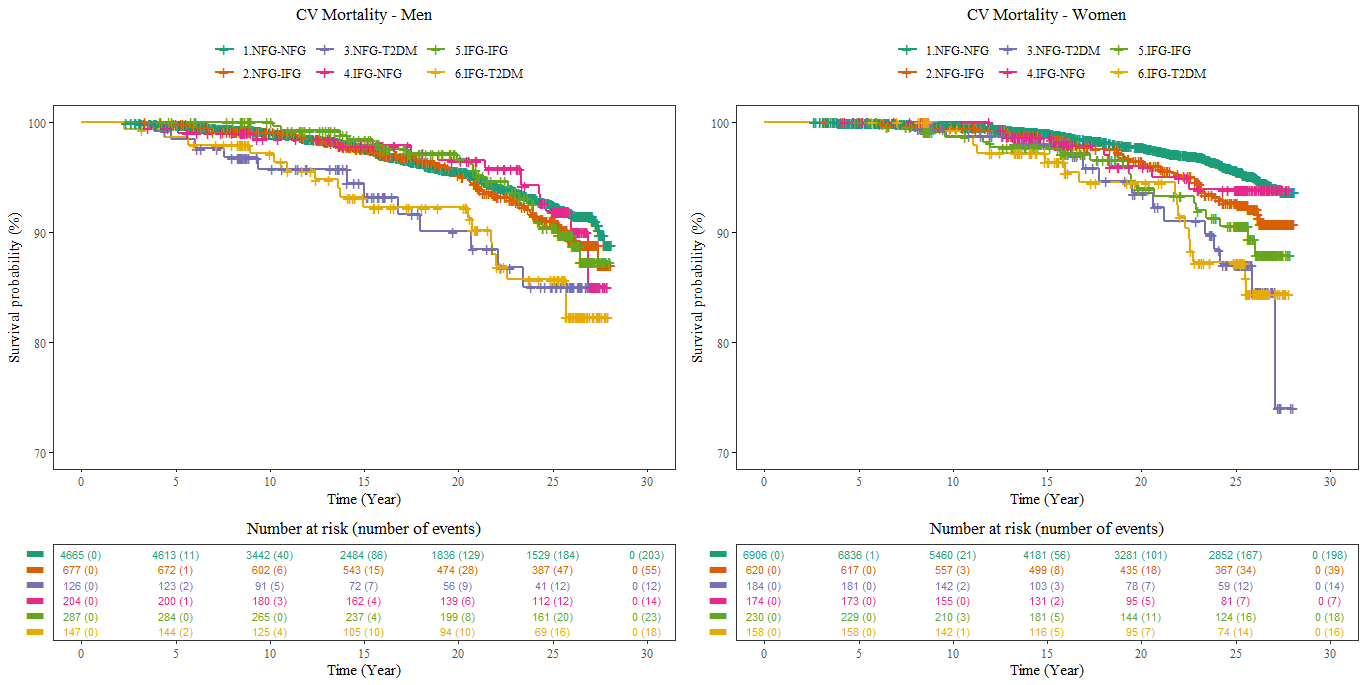


**Figure S3.** Kaplan-Meier curves for CV mortality stratified by six categories of FPG changes, separately in men and women.

CV: cardiovascular; FPG: fasting plasma glucose; NFG: normal fasting glucose; IFG: impaired fasting glucose (based on ADA cut-off (100 mg/dl)); T2DM: type 2 diabetes mellitus


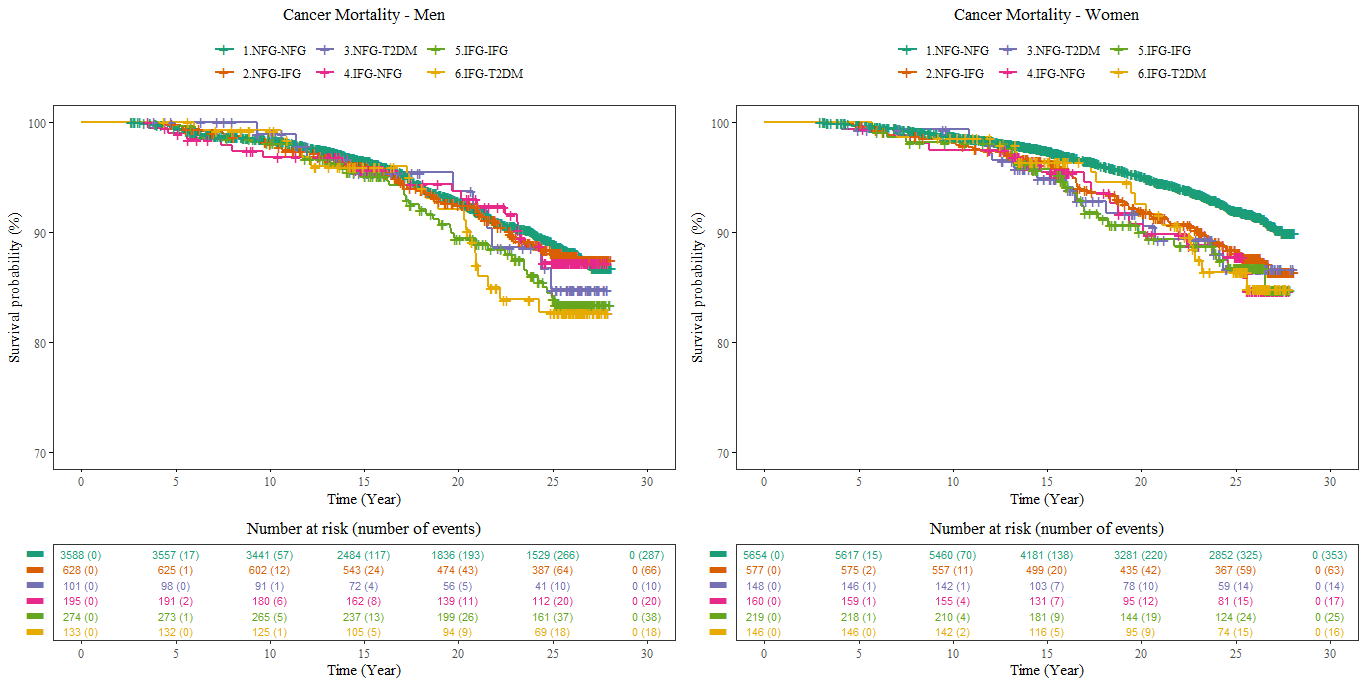


**Figure S4.** Kaplan-Meier curves for cancer mortality stratified by six categories of FPG changes, separately in men and women.

FPG: fasting plasma glucose; NFG: normal fasting glucose; IFG: impaired fasting glucose (based on ADA cut-off (100 mg/dl)); T2DM: type 2 diabetes mellitus

Only ARIC and TLGS participants were used and followed up for cancer mortality outcomes.

**References:**

1. Investigators A. The Atherosclerosis risk in COMMUNIT (ARIC) study: design and objectives. American journal of epidemiology. 1989;129(4):687-702.

2. Bild DE, Bluemke DA, Burke GL, Detrano R, Diez Roux AV, Folsom AR, et al. Multi-ethnic study of atherosclerosis: objectives and design. American journal of epidemiology. 2002;156(9):871-81.

3. Azizi F, Zadeh-Vakili A, Takyar M. Review of rationale, design, and initial findings: Tehran Lipid and Glucose Study. International journal of endocrinology and metabolism. 2018;16(4 Suppl).
